# Tonsillar Extirpation

**Published:** 1914-01

**Authors:** 


TONSILLAR EXTIRPATION.—Dr. Gottfried Trautman in a paper on the "Technic of Extra Capsular Total Extirpation of the Tonsils" (*Muench. med. Woch.*, Oct. 7, 1913), says in describing his method of anesthetization, "I object to general narcosis as a rule, as the operation can be done absolutely painlessly under local anesthesia within a very short time. I paint the tonsillar region with a ten per cent cocain solution, then inject 5 cc. of a two per cent novocain-suprarenin solution underneath the epithelium, using a curved needle. This is injected into various parts, one into the soft palate above the posterior arc, then at three points in the posterior palate arc from above downwards, the same manner into the anterior palate arc and finally into the glosso-palatine junction. Tonsil and praeputium remain untouched. The injected area must present itself like a large pemphigus. After 5 minutes, sensation begins to disappear, and soon becomes complete."

